# Deep Learning-based Design of Peptide Binders

**DOI:** 10.2533/chimia.2026.265

**Published:** 2026-04-29

**Authors:** Martin Pacesa

**Affiliations:** Institute of Pharmacology & Toxicology, University of Zurich, Winterthurerstrasse 190, CH-8057 Zurich

**Keywords:** Binders, Deep learning, Peptide design, Therapeutics

## Abstract

Peptides are short chains of amino acids that naturally mediate diverse biological functions, such as intercellular signalling, immune modulation, antimicrobial defence, and others. In recent years, peptides have also emerged as an attractive therapeutic modality. They combine best features of small molecules and biologics, enabling binding to previously undruggable molecular surfaces while remaining compact and amenable to scalable manufacturing. Yet designing peptide binders remains challenging because their interfaces are small, solvent exposed, and often highly dynamic. Recent advances in deep learning have begun to make this problem more tractable by enabling target-conditioned generation and prioritisation of peptide candidates at scale.

## 1 What are Peptides and Why are They Interesting

Firstly, by ‘peptide’ I refer to polypeptides shorter than 30 amino acids, to distinguish them from somewhat larger ‘miniproteins’ (40–100 amino acids), for which current computational protein design pipelines are generally developed.^[1]^ This cut-off is arbitrary, but I nevertheless find it useful because around this length we start to observe properties that make peptides unique and inherently more challenging to design as binders. These molecules often lack a stable folded state in isolation, present only a limited interaction surface, and can no longer rely on the same degree of shape complementarity and distributed binding energy that larger proteins or antibodies routinely exploit.^[2]^

Peptides naturally function as signalling ligands, hormones, neurotransmitters, antimicrobial effectors, trafficking motifs, toxins, and recognition elements.^[3]^ Their utilisation across the tree of life highlights their evolutionary value as ‘cheap innovators’ because short, flexible sequences can arise readily, be tuned by a small number of mutations, diffuse and turn over rapidly, and engage diverse targets through conformational plasticity.^[4]^ From a therapeutic perspective, peptides occupy a particularly attractive middle ground between small molecules and larger biologics. Small molecules primarily target hydrophobic pockets but struggle on broad or featureless protein surfaces.^[5]^ Antibodies, by contrast, can bind most targets with excellent affinity and specificity, but are costly to produce and limited to mainly extracellular applications.^[6]^

Peptides can bridge this gap. They give us more atoms to work with and are large enough to engage extended protein surfaces, yet small enough to be chemically synthesised in a scalable way. They can also be tuned through cyclization, stapling, backbone modification, terminal capping, PEGylation, lipidation, and incorporation of noncanonical amino acids.^[3]^ Such modifications can drastically improve their protease resistance, conformational preorganization, pharmacokinetics, permeability, and selectivity.^[3]^ This shows how the peptide space can be expanded both chemically and structurally far beyond simple linear amino acid chains.

## 2 Properties and Challenges of Peptide Binders

**Fig. 1:**
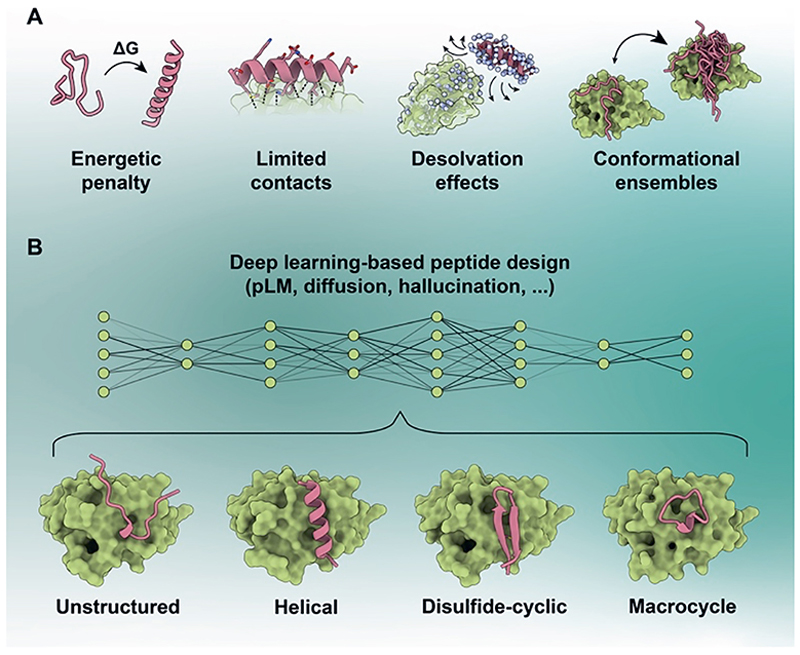
Deep learning can help overcome some traditional challenges in the design of peptide binders. (A) Peptide binders face several intrinsic challenges compared with larger protein binders, including smaller interaction surfaces, entropic penalties associated with folding upon binding, stronger competition with solvent due to limited burial, and the prevalence of dynamic binding ensembles. Most of these biophysical constraints are not explicitly accounted for in present pipelines. (B) Deep learning pipelines can generate peptide binders to arbitrary targets from sequence or structural representations, producing diverse binding geometries.

Conceptually, peptide binder design is fundamentally harder than design of larger protein binders for several reasons (Fig. 1A). The first is simply the modality and interface size. Peptides have less surface area to form favourable molecular interactions, so each contact matters more and small modelling errors carry a larger relative energetic penalty.^[2]^ The second aspect is entropy. Many peptides are not strongly preorganized in the unbound state, meaning that productive binding must pay a substantial conformational penalty.^[7]^ The third is solvent exposure. Because peptide interfaces bury less surface area, solvent interactions compete more strongly with intermolecular contacts, meaning that each individual interaction contributes more critically to binding stability. Finally, peptide binding is frequently dynamic. What matters may not be one rigidly bound structure, but an ensemble of partially preorganized conformations, transient complexes, and induced-fit rearrangements.^[8]^ Current design pipelines do not currently account for this and tend to reduce this problem to a single predicted complex and a handful of confidence metrics.

Additionally, peptide binders can come in different forms (Fig. 1B). Some bind as amphipathic helices, others as β-hairpins, extended strands, or structured loops.^[2]^ Many can be largely disordered on their own and only become ordered upon binding.^[2]^ Some peptides can depend on rigidified cyclic or disulfide-constrained topologies to reduce entropic costs,^[3]^ while others can remain at least partly fuzzy even in the bound state.^[8]^ This diversity is often underappreciated in discussions of peptide design. ‘Peptide binder’ is not a single structural class, and a design framework that performs well on helical binders may fail on fuzzy interactors or on compact macrocycles. That heterogeneity is one reason why the field has advanced slower than one might have expected from recent successes in protein design more broadly.

Traditionally, peptide binders have been discovered by display or library-based screening approaches or mined from natural sources. These approaches can be effective, but they offer limited control over binding mode and are not always straightforward to optimize beyond the initial hit. More fundamentally, purely empirical screening is an inefficient way to traverse an enormous and chemically diverse space when the properties we care about, such as affinity and specificity, are highly target-, geometry-, and system-dependent. Computational design offers a more attractive route because it can bias the search toward peptides that are already shaped for a given surface, binding mode, or biochemical and pharmaceutical properties. The real promise is not to replace experiments, but to make experimental screening smaller, more informed, and more rational from the outset.

This is where deep learning has changed the landscape. Previously, peptide design was constrained because candidate peptides had to be placed in a realistic binding geometry and then scored with enough accuracy to separate true binders from the overwhelming number of decoys.^[9]^ Deep learning changed this by learning transferable constraints on peptide-protein recognition from the data distribution of protein structures and sequence co-evolution, which makes it easier to generate plausible designs. Even so, reliably identifying true binders remains a central challenge, and many methods still rely on imperfect prediction and filtering oracles.^[10]^

## 3 Validated Pipelines for Peptide Binder Design

One emerging direction relies primarily on protein language models (pLMs), which learn latent representations of protein sequences from large sequence databases. These models capture patterns related to residue compatibility, motif usage, and aspects of structure and interaction propensity directly from sequence statistics.^[11]^ As a result, they can be used to generate peptide candidates conditioned on a target sequence without explicitly requiring structural information. Methods such as PepMLM^[12]^ and PepPrCLIP^[13]^ exemplify this approach by generating peptide sequences conditioned on a target and learned co-evolutionary signatures. This is appealing because it does not require an explicit target structure and can therefore be applied to targets that are unstructured or conformationally heterogeneous. However, sequence-only design still relies on interaction features that are implicitly encoded in the model and lacks an explicit representation of the peptide-target interaction context, which can make it difficult to reliably identify true binders.

A second class of approaches addresses this limitation more directly by generating peptide binders compatible with a target interface using structural information. One direction uses diffusion-based generative models, which sample peptide backbones directly in three-dimensional space conditioned on a target structure. An early example is RFpeptides,^[14]^ which adapts RFdiffusion^[15]^ to generate macrocyclic peptide scaffolds geometrically compatible with a target interface followed by sequence design. Using this approach, peptide binders were obtained against several protein targets including MCL1, MDM2, GABARAP and RbtA. The macrocycles exhibited affinities ranging from 6 nM to 2 μM and exceptional structural agreement between designs and crystal structures.^[14]^ More recent frameworks extend this concept to broader peptide binder modalities. For example, BoltzGen integrates structure prediction and generative modelling in an all-atom framework capable of designing multiple binder types, including linear peptides and disulfide-bonded cyclic peptides.^[16]^ In experimental campaigns, peptide binders were obtained against RagC and the RagA:RagC complex, yielding multiple hits after testing tens of designs, with the best linear peptide exhibiting a 3.5 μM affinity and the best macrocycle approximately 80 μM.

A more widely applied peptide design strategy relies on ‘hallucination’ or predictor-guided optimization. Here, structure prediction networks are used iteratively as design oracles to search the sequence-structure space for peptide-target complexes that satisfy predefined loss function constraints (such as prediction confidence, interface contacts, *etc*.).^[17]^ AfCycDesign represents an early adaptation of AlphaFold2^[18]^ for cyclic peptide design.^[19]^ Using a cyclic residue offset, the pipeline can effectively model macrocycle structures that can be used to engineer binders to targets such as MDM2 and Keap1 with reported inhibitory activity in the nanomolar to micromolar range. Related approaches such as EvoBind^[20–22]^ and EvoBind2^[23]^ perform joint sequence-structure searches guided by AlphaFold2 with co-evolutionary information extracted from multiple-sequence alignments and can design both linear and cyclic peptides directly using only the target sequence. EvoBind generated cyclic peptide agonists achieving an EC_50_ of 32 nM against GLP1R,^[21]^ and micromolar affinity towards the HIV envelope protein^[20]^ and RNAse A.^[23]^ Also purely structure-based hallucination pipelines with no provided co-evolutionary priors have been shown to be able to efficiently generate peptide binders. For example, BindCraft^[24]^ was shown to be able to generate helical peptide binders for the oncoprotein MDM2 and the therapeutic target WDR5 with nanomolar affinity.^[25]^ Interestingly, the helical peptides could be chemically stapled to lock their conformation and improve their binding affinity.

Finally, an emerging direction seeks to expand peptide design beyond the canonical amino acid alphabet. Most current pipelines operate within the standard twenty residues used in protein structure prediction models. Novel methods such as RareFold^[26]^ extend this framework to incorporate non-canonical amino acids, enabling design of both linear and cyclic peptides in the micromolar range containing expanded chemical building blocks and allowing exploration of interaction chemistries inaccessible to conventional protein design pipelines. Several recently developed generative frameworks (BoltzGen,^[16]^ RFdiffusion3,^[27]^ HalluDesign,^[28]^ BoltzDesign,^[29]^ and others) operate at an all-atom level or allow arbitrary chemical constraints and are therefore, in principle, capable of incorporating non-canonical residues, although this capability has not yet been experimentally demonstrated.

## 4 Filling the Gaps

Overall, I think the field is at an exciting but still developing stage. Deep learning has unquestionably made peptide binder design more accessible and productive than it was even a few years ago. We now have credible examples of computational methods being capable of generating experimentally validated linear and cyclic binders. Yet peptide affinities and hit rates are still lower than what we have come to expect for larger and more structured binders. This is unsurprising, as current prediction models and downstream filtering oracles still underperform on smaller interfaces, highly flexible binders, or peptides with non-canonical components.^[26,30]^ In my view, this is now the central bottleneck.

A key limitation is that most current pipelines do not explicitly model the physical determinants of peptide-protein interactions. Instead, they rely on proxy metrics derived from structure prediction models, such as prediction confidence, interface contacts, or geometric compatibility, to rank candidate complexes. While these signals can enrich for plausible binders,^[31]^ they do not explicitly account for the thermodynamics or kinetics of binding, nor for the energetic balance between bound and unbound states. Important contributions such as solvent competition, entropic penalties associated with peptide folding, and conformational heterogeneity are therefore largely ignored.

I therefore suspect that the next phase of progress will not come simply from scaling or further optimizing existing pipelines, but from fundamentally reconsidering what these models are trained to represent. Improved representations of peptide chemistry and conformational flexibility will likely be necessary to capture the diversity of interactions that short polypeptides can form. This may include models that better represent ensembles of peptide conformations rather than single static structures, incorporate solvent and environmental effects more explicitly, and expand beyond the canonical amino acid alphabet. Advances along these directions could allow future design frameworks to more faithfully learn the physical principles governing peptide-protein recognition and ultimately improve both hit rates and achievable affinities.
